# GPS tracking data of Western marsh harriers breeding in Belgium and the Netherlands

**DOI:** 10.3897/zookeys.947.52570

**Published:** 2020-07-08

**Authors:** Tanja Milotić, Peter Desmet, Anny Anselin, Luc De Bruyn, Nico De Regge, Kjell Janssens, Raymond Klaassen, Ben Koks, Tonio Schaub, Almut Schlaich, Geert Spanoghe, Filiep T’Jollyn, Joost Vanoverbeke, Willem Bouten

**Affiliations:** 1 Research Institute for Nature and Forest (INBO), Havenlaan 88/73, 1000, Brussels, Belgium Research Institute for Nature and Forest Brussels Belgium; 2 Evolutionary Ecology, University of Antwerp, Universiteitsplein 1, 2610, Wilrijk, Belgium University of Antwerp Wilrijk Belgium; 3 Dutch Montagu’s Harrier Foundation, Postbus 46, 9679, ZG Scheemda, The Netherlands Dutch Montagu’s Harrier Foundation Scheemda Netherlands; 4 Conservation Ecology Group, Groningen Institute of Evolutionary Life Sciences, University of Groningen, Postbus 11103, 9700, CC Groningen, The Netherlands University of Groningen Groningen Netherlands; 5 Institute for Biodiversity and Ecosystem Dynamics (IBED), University of Amsterdam, Science Park 904, 1098, XH, Amsterdam, The Netherlands University of Amsterdam Amsterdam Netherlands

**Keywords:** Animal movement, bird tracking, biologging, *Circus aeruginosus*, GPS tracking, habitat use, LifeWatch, machine observation, migration data, Movebank, UvA-BiTS

## Abstract

In this data paper three datasets are described containing GPS tracking and acceleration data of Western marsh harriers (*Circus aeruginosus*) breeding in Belgium and the Netherlands. The Western marsh harrier is included as a threatened bird species in Annex I of the European Bird Directive due to the steep decline in population densities. In order to collect data of habitat use and migration behaviour, Western marsh harriers were equipped with light-weight solar powered GPS trackers developed by the Institute for Biodiversity and Ecosystem Dynamics (IBED) at the University of Amsterdam (University of Amsterdam Bird Tracking System, UvA-BiTS). These trackers automatically collect and store data on the bird’s activity and 3D position in time and transmit these data to ground stations.

The datasets were collected by the Research Institute for Nature and Forest (INBO) and the Dutch Montagu’s Harrier Foundation. Tracked Western marsh harriers were breeding in the northeast of the Dutch province of Groningen and on the opposite side of the river Ems in Germany (H_GRONINGEN), in the region of Waterland-Oudeman near the Belgian-Dutch border (MH_WATERLAND), and at the left bank of the Scheldt estuary, close to the Belgian-Dutch border and north of the city of Antwerp (MH_ANTWERPEN). Most individuals remained within 10 km from their nesting sites during the breeding season and wintered in West Africa. H_GRONINGEN contains 987,493 GPS fixes and 3,853,859 acceleration records of four individuals since 2012. MH_WATERLAND contains 377,910 GPS fixes of seven individuals. Sampling in this region began in 2013. Three more Western marsh harriers were tagged in the Scheldt estuary near Antwerp more recently in 2018 (one individual) and 2019 (two individuals) for the MH_ANTWERPEN study, which contains 47,917 GPS fixes and 227,746 acceleration records.

The three Western marsh harrier datasets were published as separate studies in Movebank (https://www.movebank.org) and archived as data packages in Zenodo (https://www.zenodo.org) to ensure long-term preservation and versioning of the data.

## Data published through

Koks B, Schlaich A, Schaub T, Klaassen R, Anselin A, Desmet P, Milotic T, Janssens K, Bouten W (2019) H_GRONINGEN – Western marsh harriers (*Circus aeruginosus*, Accipitridae) breeding in Groningen (the Netherlands). Dataset. https://doi.org/10.5281/zenodo.3552507

Anselin A, Desmet P, Milotic T, Janssens K, T’Jollyn F, De Bruyn L, Bouten W (2019) MH_WATERLAND – Western marsh harriers (*Circus aeruginosus*, Accipitridae) breeding near the Belgium-Netherlands border. Dataset. https://doi.org/10.5281/zenodo.3532940

Spanoghe G, Desmet P, Milotic T, Janssens K, De Regge N, Vanoverbeke J, Bouten W (2019) MH_ANTWERPEN – Western marsh harriers (*Circus aeruginosus*, Accipitridae) breeding near Antwerp (Belgium). Dataset. https://doi.org/10.5281/zenodo.3550093

## Rationale

The Western marsh harrier (*Circus aeruginosus* Linnaeus, 1758) is a large harrier species native to temperate and subtropical Eurasia and Africa. Due to the steep population decline observed in Europe since the 1970s, the species has been included as a threatened species in annex I of the European Birds Directive in 1979. In Flanders, the Western marsh harrier appears as an endangered species on the red list of breeding bird species (Devos et al. 2016). In the Netherlands, the Western marsh harrier is not listed as a red list species (van Kleunen et al. 2017), but breeding populations are in decline since 1990 due to similar pressures as in Flanders (changed land-use, agricultural practices etc.) (van Bruggen et al. 2011).

In 2012, the Dutch Montagu’s Harrier Foundation (GKA) initiated a GPS tracking study in the northeastern part of the Netherlands (Groningen) using lightweight, solar powered GPS tags. The research objectives for this monitoring study were to determine habitat use of Western marsh harriers in agricultural landscapes, to reveal their migration behaviour, and to study flying behaviour in the vicinity of wind turbines for estimating collision risks.

The Research Institute for Nature and Forest (INBO) started studying the ecology of the Western marsh harrier in Belgium in 2011. One of the aims was to study detailed habitat use and migration patterns. In 2013, the INBO started a GPS sensor network for birds as part of the Belgian contribution to the LifeWatch observatory, using the same technology as GKA. In this network, individuals of a breeding population of Western marsh harriers in the northern part of Flanders (Waterland-Oudeman region) were equipped with GPS trackers in collaboration with GKA. In 2018, a third population was tagged with GPS trackers in the Scheldt estuary north of the city of Antwerp. The research objectives of these projects were to study the trade-off between migratory behaviour, reproductive performance and survival, and to study the home-range area, habitat preference, and foraging behaviour of Western marsh harriers in agricultural areas. To allow greater use of the data beyond our research questions, all data are now published as open data.

## Taxonomic coverage

**Figure 1.**
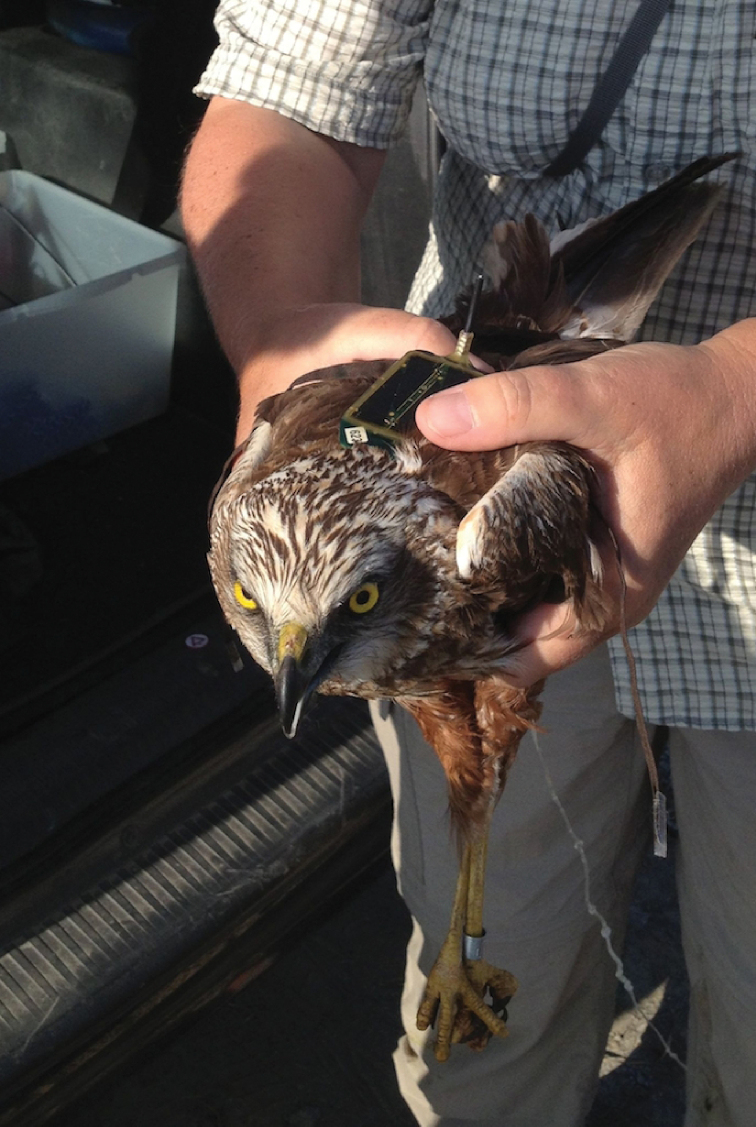
INBO researcher Anny Anselin holding Peter (animal ID L143457), one of the tagged Western marsh harriers in the MH_WATERLAND dataset (tag ID 623).

The dataset contains data from four individuals breeding in Groningen (The Netherlands) (H_GRONINGEN), seven individuals breeding near the Belgian-Dutch border (MH_WATERLAND), and three individuals breeding near Antwerp (Belgium) (MH_ANTWERPEN) (Figure 1).

### Taxonomic ranks

**Kingdom**: Animalia

**Phylum**: Chordata

**Class**: Aves

**Order**: Accipitriformes

**Family**: Accipitridae

**Genus**: *Circus*

**Species**: *Circus aeruginosus* (Linnaeus, 1758)

## Geographic coverage

**Figure 2.**
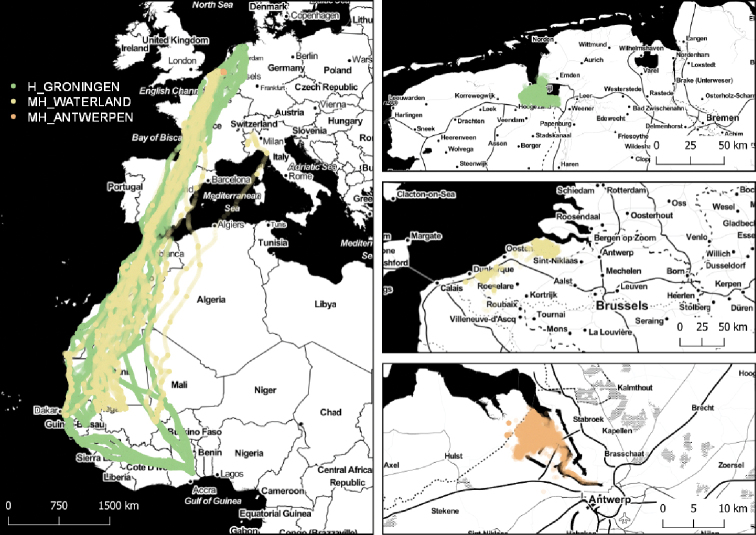
Left: Map giving an overview of the extent of the three datasets including the winter migration tracks; top right: summering data in H_GRONINGEN; middle right: summering data of MH_WATERLAND; and bottom right: summering data in MH_ANTWERPEN.

The tracked birds were breeding in the northeast of the Dutch province of Groningen and on the opposite side of the river Ems in Germany (H_GRONINGEN), in the region of Waterland-Oudeman near the Belgian-Dutch border (MH_WATERLAND), and at the left bank of the Scheldt estuary close to the Belgian-Dutch border and north of the city of Antwerp (MH_ANTWERPEN). All individuals from which data from the non-breeding period were available wintered in West Africa (Figure 2).

### Bounding box

H_GRONINGEN: 6.65N to 53.40N; 16.92W to 7.32E

MH_WATERLAND: 13.38N to 51.47N; 17.13W to 10.01E

MH_ANTWERPEN: 51.23N to 51.35N; 4.18E to 4.39E

## Temporal coverage

H_GRONINGEN: 2012-05-10 – 2018-07-11

MH_WATERLAND: 2013-05-16 – ongoing

MH_ANTWERPEN: 2018-07-18 – ongoing

## Methodology

### Study extent description

The studied Western marsh harrier populations breed in agricultural landscapes in the northeast of the Dutch province of Groningen (H_GRONINGEN; 53.278N, 6.981E), the polder area in the north-western part of Belgium (MH_WATERLAND; 51.276N, 3.595E), and in the polder area at the left bank of the Scheldt estuary in the northern part of Belgium (MH_ANTWERPEN; 51.312N, 4.285E). The tracked birds nested on the ground in small reed beds and cereal fields.

The harriers were trapped using a noose-trap on a sitting pole (Gartshore 1978) in the vicinity of their nesting place (H_GRONINGEN and MH_WATERLAND) or a bal-chatri (Berger and Mueller 1959) with live birds (MH_ANTWERPEN). As trapping proved very difficult, only a few individuals could be tagged per breeding season (Table 1). Once captured, biometrics were taken from all captured harriers: tarsus length, wing length, body mass and moulting stage following the methods of Bijlsma 1997 (H_GRONINGEN and MH_WATERLAND) and Ginn and Melville 1983 (MH_ANTWERPEN). Sex was determined on sight. UvA-BiTS GPS-trackers (Bouten et al. 2013) were attached to the birds with the body loop attachment method using a harness of Teflon tape (Figure 1).

In total, 14 Western marsh harriers were tracked (Table 1). All four individuals in the H_GRONINGEN study are assumed dead as they were not observed anymore during one or more years. In 2018, one of the tagged harriers (Roelof) came back to his breeding grounds, but the tracker got broken and he has not been spotted again in 2019 (status unknown). In the MH_ANTWERP study, two individuals were tagged in 2019, while one animal (Suzanna) was tagged in 2018 but did not come back after the migration in 2019 (status unknown). One of the individuals (Raymond) in MH_WATERLAND was found dead in the Italian Alps in spring 2016. His tracker was reused for another male (Ben). Another individual in the MH_WATERLAND dataset (Jozef) was tagged in 2013 in his breeding area in the Waterland-Oudeman region but moved to another breeding area at the Moeren close to Veurne at 70 km from his previous breeding ground in 2016, 2017 and 2018. The other individuals in this dataset have not been seen in their original breeding grounds in the past few years and are assumed dead.

**Table 1.** Overview of the tracked individuals per project, their status in 2019, total number of tracking days, number of GPS fixes and biometric data.

Project	Animal id	Animal name	Sex	Date first observation	Date last observation	Status in 2019	Tracking days	GPS fixes	Body mass (g)	Tarsus length (mm)	Wing length (mm)	Moult score
H_GRONINGEN	5327085	Job	male	2016-06-18	2016-09-03	assumed dead	78	21,337	513	70	383	0000000000
H_GRONINGEN	5336455	Kjell	male	2014-06-04	2014-06-23	assumed dead	20	5,420	540	74	406	5200000000
H_GRONINGEN	5325667	Roelof	male	2014-07-04	2018-07-11	unknown; tracker broken	1,469	781,906	504	69	418	0000000000
H_GRONINGEN	5446465	William	male	2012-05-10	2016-08-11	assumed dead	1,380	178,830	524	72	397	
MH_WATERLAND	H185298	Almut	female	2016-06-03	2016-06-13	assumed dead	11	475	656		420	
MH_WATERLAND	L143472	Ben	male	2016-05-02	2017-07-15	assumed dead	440	85,924	571		404	
MH_WATERLAND	L143451	Jozef	male	2013-06-25	2018-07-28	unknown	1,854	183,985	512	64	402	
MH_WATERLAND	H173481	Mia	female	2013-05-16	2013-08-02	assumed dead	78	13,209	785	77	430	
MH_WATERLAND	L143457	Peter	male	2013-07-22	2014-09-01	assumed dead	407	62,297	482	65	392	
MH_WATERLAND	L143467	Raymond	male	2015-05-26	2016-03-25	found dead in March 2016	305	31,070	472	71	385	
MH_WATERLAND	L143473	Walter	male	2016-06-01	2016-06-08	assumed dead	8	950	485		395	
MH_ANTWERPEN	H197169	Lilla	female	2019-04-18	2019-07-30	alive	104	28,181	810	80	426	0
MH_ANTWERPEN	L177801	Lillo	male	2019-05-16	2019-05-19	alive	4	17,046	520	72	410	0
MH_ANTWERPEN	H171693	Zuzanna	female	2018-07-18	2018-07-27	unknown	10	2,690	674	77	410	29

### Sampling description

Harriers in the three studies were equipped with the University of Amsterdam Bird Tracking System (UvA-BiTS) developed by the Institute for Biodiversity and Ecosystem Dynamics (IBED) at the University of Amsterdam. These lightweight, solar powered GPS trackers automatically record 3D position and air temperature. The built-in tri-axial accelerometer can be configured to collect body movements and bird behaviour and was deployed in the H_GRONINGEN and MH_ANTWERPEN studies. Each individual tri-axial accelerometer measurement consists of x (acceleration-raw-x), y (acceleration-raw-y) and z data points (acceleration-raw-z). Tilt values (tilt-x, tilt-y and tilt-z) are derived from the raw acceleration measurements (M), the calibration factors offset (O) and sensitivity (S).Thus acceleration for heave (tilt-z), surge (tilt-x) and sway (tilt-y) is calculated as: A_z_ = (M_z_-O_z_)/S_z_ ; A_x_ = (M_x_-O_x_)/S_x_; A_y_ = (M_y_-O_y_)/S_y_ (UvA-BiTS 2018). Tilt data are expressed in g. Both raw acceleration data and derived tilt data are collected in groups of 20 samples. These samples should be analysed as a group because these multiple data recordings are collected in a rapid sequence (up to 20 tri-axial measurements per second) to produce a complete picture of bird behaviour.

Data are stored in the tracker’s 4 MB built-in flash drive. Depending on the settings, up to 60,000 GPS records can be stored in the internal memory (Bouten et al. 2013). Trackers are equipped with a ZigBee transceiver and a whip antenna for transmitting data to a base station and for receiving new measurement settings. Unlike other bird tracking studies using similar technology (e.g., Stienen et al. 2016), base stations were not set up on fixed locations as breeding sites varied between years. Once the tagged harriers were spotted in their breeding locations, mobile base stations were used to read out data. This implies that data from birds that do not return to their previous breeding grounds cannot be retrieved unless they are spotted in a new breeding location (as happened with Jozef who moved to another breeding location in spring 2016).

Different intervals between successive GPS fixes were applied, ranging from 3 s to 30 min during the day, and 4 s to 2 h at night. In the H_GRONINGEN study, “high-resolution” GPS data with an interval of 3 s were collected during parts of the day using hourly blocks or virtual geographic fences in order to increase the positional accuracy of the GPS fixes (Bouten et al. 2013).

Data received by the base stations are automatically harvested, post-processed, and stored in a central PostgreSQL database at UvA-BiTS (http://www.uva-bits.nl/virtual-lab), which is accessible to the involved researchers only. In order to make our data available to the whole scientific community, all tracking data are eventually published as open data. We decided to upload the data to Movebank (https://www.movebank.org) as it is a specific repository for this type of data and it is well adopted by the scientific community (Mrozewski 2018). The Movebank data model enables the description of animals, tags, deployments, detections, and other measurements, such as acceleration data (Kranstauber et al. 2011).

**Figure 3.**
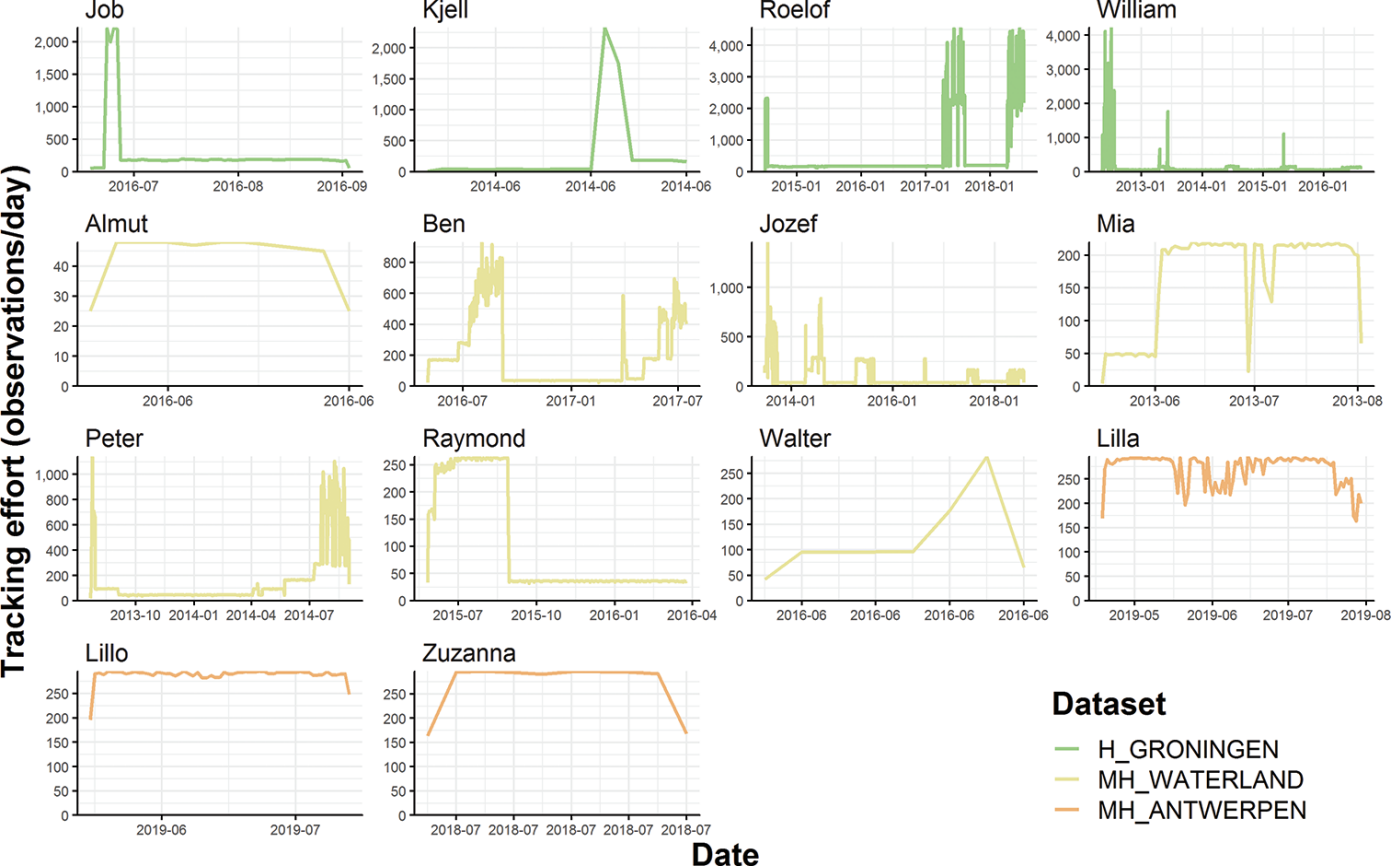
Tracking effort: number of observations per day and per individual.

Both reference, GPS data and acceleration data of our Western marsh harrier studies were downloaded from the UvA-BiTS database using SQL queries and then transformed into the Movebank data format (Movebank 2019) using R scripts (https://github.com/inbo/bird-tracking). This allows us to repeat the process when new data become available for active studies. These data were then uploaded to the Movebank database, with one study for each dataset (Table 2). As the Movebank data repository (https://www.datarepository.movebank.org/, offered as a service to archive movement data) currently does not support versioning and version-agnostic DOIs, we opted to archive our studies in the Zenodo data repository (https://www.zenodo.org). For each Movebank study, one Zenodo data package has been created (Table 2). These data packages consist of four different file types: a readme file with the terms of use and attributes of the data files, a reference data file about animals, tags and deployments, GPS data files, and files containing acceleration data. GPS and acceleration data are split into separate csv files per year, which makes it easier to download data in manageable chunks and to update these data packages with observations from an extra year. For this reason, the MH_ANTWERPEN dataset contains more GPS data records in the Movebank study compared to the Zenodo archive as data from 2019 are incomplete and will be archived on Zenodo in the course of 2020 after birds have returned from their wintering area. No GPS data are available for 2019 from birds in the H_GRONINGEN and MH_WATERLAND studies, as none of the tagged individuals were observed in 2019 (Figure 3).

**Table 2.** Datasets and the respective links to the Movebank studies and Zenodo data packages.

Title	Movebank study ID	Zenodo
H_GRONINGEN – Western marsh harriers (*Circus aeruginosus*, Accipitridae) breeding in Groningen (the Netherlands)	922263102	https://doi.org/10.5281/zenodo.3552507
MH_WATERLAND – Western marsh harriers (*Circus aeruginosus*, Accipitridae) breeding near the Belgium-Netherlands border	604806671	https://doi.org/10.5281/zenodo.3532940
MH_ANTWERPEN – Western marsh harriers (*Circus aeruginosus*, Accipitridae) breeding near Antwerp (Belgium)	938783961	https://doi.org/10.5281/zenodo.3550093

### Quality control description

GPS fixes that are likely incorrect (i.e., outliers) are marked in two ways: manually by the researcher in the UvA-BiTS database (indicated as TRUE in manually-marked-outlier) and automatically before uploading to Movebank for GPS-fixes with speeds above 30 m/s (indicated as TRUE in import-marked-outlier). Using this approach, 376, 97 and 16 observations were marked as outliers in H_GRONINGEN, MH_WATERLAND and MH_ANTWERPEN respectively. The workflow and scripts for querying data from the UvA-BiTS database and transforming these into the Movebank data format are publicly documented on GitHub (https://github.com/inbo/bird-tracking).

### Method step description

Data recording

1. Researcher captures bird, takes biometrics, attaches GPS tracker, and releases bird.

2. Researcher records or updates metadata about bird, GPS tracker and deployment.

3. Researcher sets a measurement scheme, which can be updated anytime.

4. GPS tracker records data.

5. GPS tracker automatically receives new measurement settings and transmits recorded data when a connection can be established with the mobile base station.

6. Recorded data are automatically harvested, post-processed, and stored in a central PostgreSQL database at UvA-BiTS.

7. Data stream stops when birds no longer return to the nesting site or if GPS trackers no longer function.

Data publication

1. Data (reference, GPS and acceleration) are periodically exported from UvA-BiTS in the Movebank data format.

2. GPS outliers are marked.

3. Data are uploaded to the appropriate study on Movebank and made publicly available.

4. Data are exported from Movebank and archived on Zenodo, where each update is a version with a DOI.

## Datasets

### Dataset description

**Figure 4.**
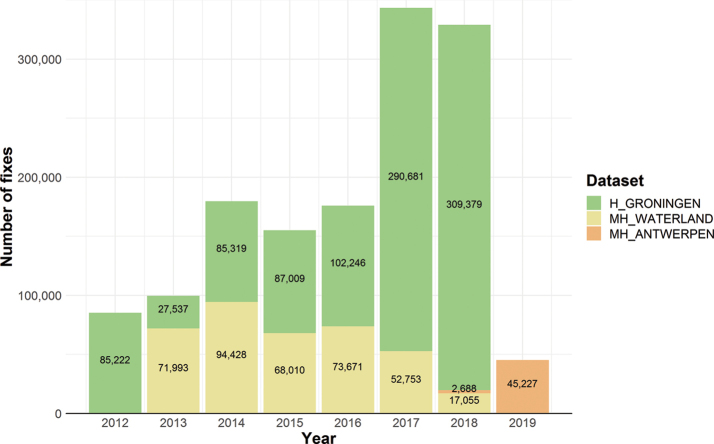
Number of GPS fixes per year and per dataset.

**Figure 5.**
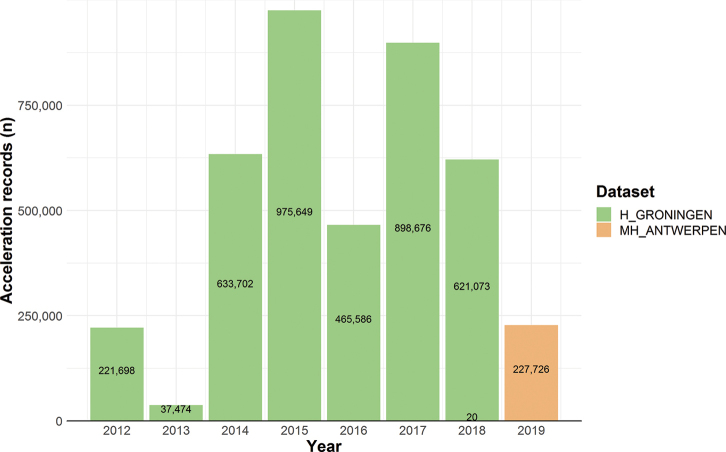
Number of acceleration records per year and per dataset.

Our data are grouped in three datasets (one dataset per study area). H_GRONINGEN is the largest dataset containing 987,493 GPS fixes in the period 2012–2018, while the MH_WATERLAND study started in 2013 with 377,910 GPS fixes until 2018, and MH_ANTWERPEN started in 2018 and contains 47,917 GPS fixes in the Movebank study for the period 2018–2019 (Figure 4). In the H_GRONINGEN and MH_ANTWERPEN studies acceleration data were collected as well, with respectively 3,853,859 and 227,746 acceleration records (Figure 5).

**H_GRONINGEN dataset**

• **Object name**: H_GRONINGEN – Western marsh harriers (*Circus aeruginosus*, Accipitridae) breeding in Groningen (the Netherlands)

• **Format name**: Movebank data format

• **Format version**: 2 (http://vocab.nerc.ac.uk/collection/MVB/2/)

• **Language**: English

• **License**: http://creativecommons.org/publicdomain/zero/1.0/

• **Usage norms**: http://www.inbo.be/en/norms-for-data-use

• **Publication date**: 2019-11-26

• **Derived from**: https://www.movebank.org/cms/webapp?gwt_fragment=page=studies,path=study922263102

• **DOI of version described in this paper**: https://doi.org/10.5281/zenodo.3828298

• **DOI for all versions**: https://doi.org/10.5281/zenodo.3552507

**MH_WATERLAND dataset**

• **Object name**: MH_WATERLAND – Western marsh harriers (*Circus aeruginosus*, Accipitridae) breeding near the Belgium-Netherlands border

• **Format name**: Movebank data format

• **Format version**: 2 (http://vocab.nerc.ac.uk/collection/MVB/2/)

• **Language**: English

• **License**: http://creativecommons.org/publicdomain/zero/1.0/

• **Usage norms**: http://www.inbo.be/en/norms-for-data-use

• **Publication date**: 2019-11-12

• **Derived from**: https://www.movebank.org/cms/webapp?gwt_fragment=page=studies,path=study604806671

• **Source of**: https://doi.org/10.15468/rbguhj (earlier version of dataset published to the Global Biodiversity Information Facility in the Darwin Core format)

• **DOI of version described in this paper**: https://doi.org/10.5281/zenodo.3826591

• **DOI for all versions**: https://doi.org/10.5281/zenodo.3532940

**MH_ANTWERPEN dataset**

• **Object name**: MH_ANTWERPEN – Western marsh harriers (*Circus aeruginosus*, Accipitridae) breeding near Antwerp (Belgium)

• **Format name**: Movebank data format

• **Format version**: 2 (http://vocab.nerc.ac.uk/collection/MVB/2/)

• **Language**: English

• **License**: http://creativecommons.org/publicdomain/zero/1.0/

• **Usage norms**: http://www.inbo.be/en/norms-for-data-use

• **Publication date**: 2019-11-21

• **Derived from**: https://www.movebank.org/cms/webapp?gwt_fragment=page=studies,path=study938783961

• **DOI of version described in this paper**: https://doi.org/10.5281/zenodo.3827918

• **DOI for all versions**: https://doi.org/10.5281/zenodo.3550093

## Usage norms

To allow anyone to use these datasets, we have released the data to the public domain under a Creative Commons Zero waiver (http://creativecommons.org/publicdomain/zero/1.0/). We would appreciate however, if you read and follow these norms for data use (http://www.inbo.be/en/norms-for-data-use) and provide a link to the original dataset using the DOI whenever possible. If you use these data for a scientific paper, please cite the dataset(s) following the applicable citation norms and/or consider us for co-authorship. We are always interested to know how you have used or visualized the data, or to provide more information, so please contact us via the contact information provided in the metadata or opendata@inbo.be.
